# Children’s Recall of Words Spoken in Their First and Second Language: Effects of Signal-to-Noise Ratio and Reverberation Time

**DOI:** 10.3389/fpsyg.2015.02029

**Published:** 2016-01-14

**Authors:** Anders Hurtig, Marijke Keus van de Poll, Elina P. Pekkola, Staffan Hygge, Robert Ljung, Patrik Sörqvist

**Affiliations:** ^1^Department of Building, Energy and Environmental Engineering, University of GävleGävle, Sweden; ^2^Linnaeus Centre HEAD, Swedish Institute for Disability Research, University of LinköpingLinköping, Sweden; ^3^Department of Education, Health and Social Studies, University of DalarnaFalun, Sweden

**Keywords:** children, speech perception, reverberation time, signal-to-noise ratio, second-language, classroom acoustics

## Abstract

Speech perception runs smoothly and automatically when there is silence in the background, but when the speech signal is degraded by background noise or by reverberation, effortful cognitive processing is needed to compensate for the signal distortion. Previous research has typically investigated the effects of signal-to-noise ratio (SNR) and reverberation time in isolation, whilst few have looked at their interaction. In this study, we probed how reverberation time and SNR influence recall of words presented in participants’ first- (L1) and second-language (L2). A total of 72 children (10 years old) participated in this study. The to-be-recalled wordlists were played back with two different reverberation times (0.3 and 1.2 s) crossed with two different SNRs (+3 dBA and +12 dBA). Children recalled fewer words when the spoken words were presented in L2 in comparison with recall of spoken words presented in L1. Words that were presented with a high SNR (+12 dBA) improved recall compared to a low SNR (+3 dBA). Reverberation time interacted with SNR to the effect that at +12 dB the shorter reverberation time improved recall, but at +3 dB it impaired recall. The effects of the physical sound variables (SNR and reverberation time) did not interact with language.

## Introduction

Noise in a classroom hampers memory and learning in at least two different ways. First, transportation noise, such as aircraft and road traffic noise, has been shown to impair learning and recall from reading a text while being noise exposed. This has been shown in laboratory studies (Hygge, 2003; Sörqvist, 2010), in field experiments (Hygge, 2003; Hygge et al., 2003) and in two large field studies on children (Hygge et al., 2002; Stansfeld et al., 2005). The findings from these two field studies were the important factors behind WHO’s decision to devote a chapter in their publication *Burden of disease from environmental noise* (WHO, 2011) to children and cognition.

The other main channel through which learning in classrooms is negatively influenced is when the speech signal from the teacher, rather than reading a text, is the material to be learnt. This speech signal can be degraded and distorted in several ways, including by a high background noise level, which makes the signal-to-noise ratio (SNR) unfavorable for listening. The speech signal can also be distorted by a too long reverberation time (e.g., 1.2 s), which degrades the speech signal by imposing echo effects. A functionally, but not acoustically, similar hampering effect will occur when the speech signal is degraded by characteristics of the listener, e.g., when the information conveyed is difficult or complicated; or when the information is presented in a language which is not the mother tongue of the listener; or when the listener does not have a high working memory capacity (WMC) or perfect hearing, which often is the case in elderly people and young children (Klatte et al., 2013; Rönnberg et al., 2013).

The acoustical properties in a classroom are often defined and measured in term of SNR and reverberation time. Several studies indicate that people can recall more from messages presented in the context of a higher SNR in comparison with a lower SNR (Kjellberg et al., 2008; Ljung et al., 2013). Similarly, shorter reverberation time appears to be favorable for learning compared to a longer reverberation time (Ljung and Kjellberg, 2009). An important aspect of the effects of classroom acoustic factors on memory and learning is not only the factor’s individual contribution to learning, but also the relative magnitude of their effects when studied in combination (i.e., interaction effects). That is, it is of practical interest to know how much a given SNR decrement can be compensated for by an improved reverberation time. Or put into a more theoretical framework: Does SNR and reverberation time interact? Under the assumption that the two factors influence the same cognitive process (e.g., if they both require the same form of cognitive compensation to reach a desirable level of speech perception), the factors should interact.

Klatte et al. (2010) investigated the effects of classroom noise and reverberation time in the context of speech perception (the ability to perceive individual words) and listening comprehension (the ability to make sense of longer discourse). They found a significant interaction between reverberation and background sounds on children’s speech perception such that reverberation had a stronger negative effect when background sounds were at a high level. Moreover, listening comprehension was more sensitive to the noise effects than speech perception, but Klatte et al. (2010) did not vary the reverberation time in their listening comprehension test. In the present study, we investigated the interaction between reverberation and SNR on a yet later stage of the speech processing chain—recall of the spoken material. Even though recall must necessarily depend on the ability to perceive what is said, these processing stages (perception, comprehension, and recall) are functionally distinguishable. Both comprehension (Klatte et al., 2010) and recall (Kjellberg et al., 2008; Hygge et al., 2015) appears to be more sensitive to noise effects than speech perception and speech intelligibility are.

Language proficiency is expected to affect any interaction between reverberation time and SNR. A listener’s capability to recall something that was said in the listeners’ first language (L1) should be easier, and perhaps less vulnerable to the effects of SNR and reverberation, than when the speech is in the listener’s second language (L2). Several studies show that people are less susceptible to the effects of noise on speech intelligibility and comprehension when the speech signal is presented in the listener’s first language (Pichora-Fuller et al., 1995; Mayo et al., 1997; Kidd et al., 2007; Tabri et al., 2011; Kilman et al., 2014; Sörqvist et al., 2014), but to the best of our knowledge, no studies have combined SNR and reverberation time to investigate effects on memory of words in different languages for school children.

The listeners WMC is yet another important factor in the context of assessing memory and recall of speech in different listening combinations of SNR and reverberation time. Individual variations in WMC predict the ability to identify (Rönnberg et al., 2013) and remember (Kjellberg et al., 2008; Sörqvist and Rönnberg, 2012; Ljung et al., 2013) masked spoken messages. High capacity individuals are more able to compensate for the signal distortion than their low capacity counterparts, perhaps because they can use contextual cues more efficiently when signal distortion makes it difficult to identify what is being said or because they can inhibit the undesired processing of the non-target (noise) information (Rönnberg et al., 2013). The predictive power of individual differences in WMC can explain why age co-varies with the ability to recognize speech in degraded conditions, as age differences typically reflect differences in WMC (Sörqvist and Rönnberg, 2014). It is less certain whether variations in WMC within a young sample would also predict the ability to recall information presented in degraded listening conditions.

The present study was designed to explore some of these issues. Wordlists in English and Swedish were presented to Swedish speaking children in grade 4 (10 years, *N* = 72). The wordlists were presented with SNRs of +3 dB and +12 dB crossed with reverberation times of 1.2 and 0.3 s. The dependent measure was free recall of the words, calculated as the probability (0–1) of recall of the words. We expected SNR and reverberation time to interact in their effects on recall, and kept the possibility open that this interaction would be different for the two languages and vary with the participants’ WMC.

## Materials and Methods

### Participants

A total of 72 children participated in the study. The children were pupils in four different classes from two different elementary schools in Gävle, Sweden. All participants were 10 years old and had normal or corrected to normal hearing and vision. As a reward for participation, the four classes were each paid 2000 SEK to spend on a collective class-activity. This study is a part of a larger project for which we have a legal ethical approval from the Regional Ethical Board in Uppsala (Nr 338/2011). The school administration gave their consent to the study, and written information about the study was sent home to the parents/guardians. Contact persons in the research group were listed and the choice not to participate was clearly stated. No family or guardian did that, or any of the pupils. However, four pupils with reading or writing problems took part in at least some part of the study but their performance on the recall test was not scored.

### Materials and Apparatus

#### Sound and Wordlists

Twenty-four wordlists with eight words per list were composed. Categories and words were chosen from an expanded version of the Battig and Montague (1969) norms (van Overschelde et al., 2004). A total of 24 categories were used to create a set of 24 unique word lists. Eight of the most common words were chosen from every category, while taking into account phonological characteristics so that every list consisted of words of about the same average number of syllables. The semantical relatedness of the chosen categories was not close to each other (e.g., if the category ‘fruit’ was chosen, ‘vegetables’ was not chosen, as they are strongly semantically related). The words used in each list were phonologically dissimilar, i.e., they did not rhyme. Each individual wordlist appeared equally often in the four combinations of SNR and reverberation time.

By means of Graeco–Latin squares the words from the 24 categories were organized into 12 Swedish and 12 English lists of words, where the average category rank order of the words was the same. After composing the English wordlists, a fourth-grade English (L2) teacher (fluent in both English and Swedish) screened the wordlists and judged the words’ applicability for fourth-grade children and some of the words were replaced by other words from the same category. After this modification the average number of syllables were about the same for the English and Swedish words (means English: 1.52, Swedish: 1.56, *F* < 1) and there was no significant interaction between Language and Position (1–8) in the list (*F* < 1). Thus, it can be ruled out that the difference in difficulty between the English and Swedish wordlists were not a mere reflection of the length of the words and the number of syllables.

Also, an independent assessment of the word lists in the experiment was made with another group of Grade 4 children (*N* = 71) who listened to the word lists in good listening conditions in their classroom. For each word they stated whether they recognized the words, scored as 1 = yes, 0.5 = more Yes than No, and 0 = No. The Swedish words had an average score of 0.94 and the English words a score of 0.82. This difference between the languages was highly significant, *F*(1,70) = 60.94, *MSE* = 0.82, $\eta_{p}^{2}$ = 0.47, *p* < 0.001.

The wordlists were recorded in an anechoic room by a bilingual female speaker, fluent in both English and Swedish. Broadband noise was added to the wordlists to create SNRs at +3 dB and +12 dB. The words were manipulated in an acoustical software program to create a long reverberation time (1.2 s) and a short reverberation time (0.3 s). To avoid an overlap between words in the long reverberation time conditions, the inter-stimulus interval, onset-to-onset, was set to three seconds. All words within the same list had the same reverberation time and the same SNR. There was no noise between the words.

The wordlists (three per experimental condition) were played back to the children through eight loudspeakers (Cambridge Audio, Minx, Min 11) that were hung from the ceiling of a classroom, the amplifier used was a Denon AVR-2113. The number of loudspeakers was chosen with the intent to create an even dB level in the classroom, regardless of the child’s positioning in the classroom. The sound level was set to approximately 66 dBA. Recall was made by pen and paper. Because language skills varied, particularly in L2, the children were instructed just to write down the words they had heard and that the correct spelling of the words was not important. After every wordlist, the children had 30 s to write down the words they could recall in free order. After these 30 s, the next wordlist was played back.

#### Children’s Size-Ordering Task

A version of the children’s size-ordering task (McInerney et al., 2005) was adapted to measure the children’s WMC. In this task, Swedish nouns were presented on a screen in front of the classroom. The words were shown one by one. Each word was visible for one second, and followed immediately by the next word. The words were representing objects of different sizes (e.g., church, television, car) and the order of the words were random at presentation. After every list, the children were supposed to write down the objects in the accurate order, from the smallest to the largest object, and the number of correctly reported relationships was recorded. There were 11 lists in total for the whole test (two lists with two words, three lists with three words, three lists with four words, and three lists with five words). The test started with the shortest lists and then increased incrementally throughout the test. The lists containing two words were only used as a training phase for the pupils to get acquainted with the task. Those lists were not included in the analyses.

### Design and Procedure

An ANOVA of the probabilities of recalling the words was performed with Language, SNR, and reverberation time as fully crossed within-person factors. The presentation order of the combinations of the independent conditions was counter balanced between participants. The children were tested as a group in their ordinary classrooms, in their regular places in the classroom, facing the front of the classroom. Each class consisted of 15–21 children. At the start, the children were informed that they were free not to participate and it was also emphasized that their teachers would not be informed about how well they performed. The test session started with the size-ordering task. This part of the experiment took about five minutes to complete. The wordlist session started after completion of the size-ordering task. The wordlist session took about 40 min to complete. The pupil’s teacher was present in the classroom during the whole experiment.

## Results

The recall of the words recalled was scored as correct (score = 1) or incorrect (score = 0). In the statistical analysis the dependent variable was the probability of recall of the words, ranging from 0 to 1. The independent within person variables were Language, SNR and reverberation time. The size ordering WMC task was an independent between person variable.

A trichotomized split of the scores in the two most difficult size ordering WMC tasks (*N*s = 23, 25, 24, Means WMC 0.52, 2.22, 5.88) revealed a significant main effect of WMC on recall, with mean probabilities of 0.23, 0.24 and 0.30 in each WMC group, respectively, *F*(2,69) = 6.55, *MSE* = 0.99, $\eta_{p}^{2}$ = 0.16, *p* < 0.010. However, there was no significant interaction between WMC and any of the other variables or their combinations (all *Fs* < 1.73). Therefore, to save on sensitivity, *p*-values, effect size, and power in all the subsequent analyses were therefore performed without the WMC variable. It should be noted, when comparing the analyses with and without the WMC-measure, only negligible differences were noted and none of them affected the pattern of significant effects. Thus, we decided not to include any analysis of the WMC-test herein.

The recall means for the combinations of Language, SNR and reverberation time are shown in **Table 1**. In passing, and without statistical testing, it can be calculated from **Table 1** that the average *absolute* difference in probability of recall when listening at +12 dB compared to +3 dB is about the same for English and Swedish (0.11 and 0.13, respectively), but also that the *relative* increment in probability is more than 90% for English and 48% for Swedish.

**Table 1:** Means probability recall by language, signal-to-noise ratio (SNR), and reverberation time.

	SNR					
	+3 dB			+12 dB		
Language	RevT = 1.2 s	RevT = 0.3 s		RevT = 1.2 s	RevT = 0.3 s	Means row
English	0.123	0.113		0.214	0.242	0.173
Swedish	0.286	0.258		0.379	0.427	0.338
Means column	0.205	0.185		0.296	0.335	0.255
Means SNR	+3 dB = 0.195			+12 dB = 0.316		

*RevT = Reverberation time*.

**FIGURE 1:**
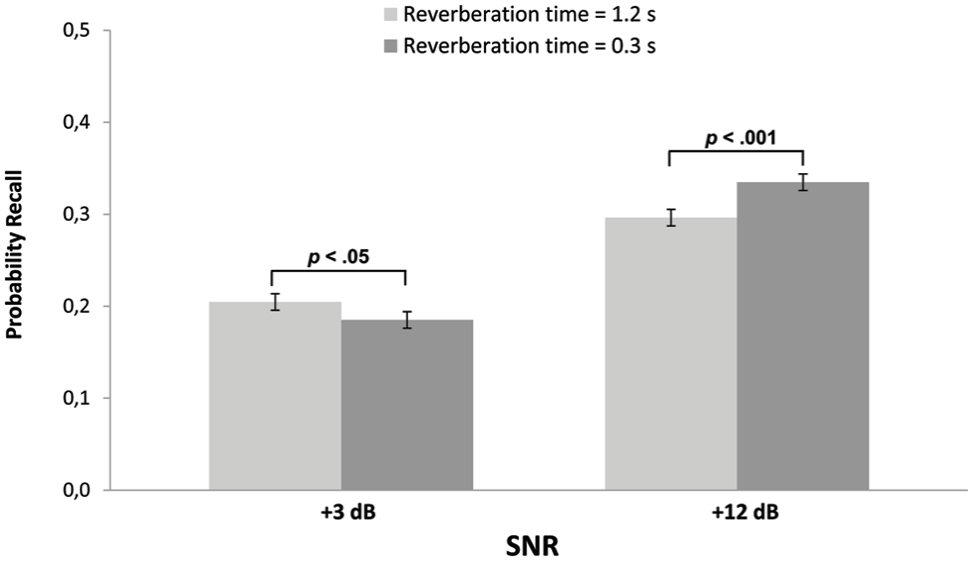
**Probability of recall as a function of signal-to-noise ratio (SNR) and reverberation time**. Error bars are the standard errors of the mean differences between the two levels of reverberation time.

**Table 2** shows the *F*-statistics for the significant effects of all the independent variables Language, SNR, reverberation time, and their interactions. The power and the effects sizes of the main effects of Language and SNR were remarkably high. The crossover interaction SNR^∗^reverberation time is illustrated in **Figure 1** which shows that the children were better at recalling the words when the words were presented with a SNR of +12 dBA instead of a SNR of +3 dBA. Moreover, reverberation had opposing effects depending on the SNR level. At +12 dB a longer reverberation time (1.2 s) impaired memory compared with a reverberation time of 0.3 s. When the SNR was +3 dB the longer reverberation time unexpectedly improved the children’s recall. For **Figure 1** tests of simple main effects of the difference between the reverberation times 1.2 and 0.3 s at the two SNR levels showed significant effects at both levels, but in opposite directions, for +3 dB *F*(1,71) = 4.25, *p* < 0.05 and for +12 dB *F*(1,71) = 17.11, *p* < 0.001.

**Table 2:** *F*-statistics for the significant effects of all the independent variables language, SNR, reverberation time, and their interactions.

Source	*SS*	*df*	*MS*	*MSE*	*F*	*p*	$\eta_{p}^{2}$	Power
Language	93.68	1	93.68	0.355	263.68	<0.001	0.79	1.00
SNR	50.32	1	50.32	0.205	245.89	<0.001	0.78	1.00
Reverberation time^∗^SNR	2.89	1	2.89	0.179	16.15	<0.001	0.19	0.98

To summarize, the results showed that (1) WMC had a main effect on recall, but did not interact with the other variables, (2) Language and SNR had strong effects on recall, and (3) there was a crossover interaction between SNR and reverberation time to the effect that at the better SNR +12 dB, the shorter (0.3 s) reverberation time impaired recall, but at SNR +3 dB and the longer (1.2 s) reverberation time improved recall.

## Discussion

The children recalled more words when the words were presented in the children’s native language, Swedish, compared to their second language, English. When SNR-ratio was high, compared to low, they also recalled more words. Moreover, recall was better in the short reverberation time condition in comparison with the long reverberation time condition, but only when the SNR was high. Conversely, when the SNR was low, recall was better when the reverberation time was longer. Hence, the results followed the predictions except for the admittedly surprising finding that reverberation time had an advantageous effect on recall when the SNR was low.

A low SNR decreases speech intelligibility. As speech intelligibility was presumably lower in the low SNR conditions, additional effort was required to identify the words in these conditions compared to conditions with high SNR (Klatte et al., 2010, 2013). This could explain why recall was impaired by lower SNR, because the additional effort would reduce the capacity to process, store and subsequently recall the words (Rabbitt, 1966; Kjellberg et al., 2008). The resources explanation could also explain why the children were less able to recall words spoken in their L2 (English), as it may have been more effortful to identify these words. An alternative explanation of this language effect is that the children were unfamiliar with the English words, but this explanation is not easily reconciled with the fact that fourth graders were familiar with over 80% of the English words (in comparison with over 90% of the Swedish words). Additional factors are probably at work. For example, it might have been harder for the children to make associations between the English words and their meanings, and additional resources might in turn have been needed for word retention.

The potential influence from floor effects is a concern in the data reported here. Previous research has shown that ten year old children have a mean digit span of about five items in “normal” conditions (without interfering background noise; Orsini et al., 1987; Elliot and Cowan, 2005). In the current experiment, there was no corresponding “normal” condition as the words were presented in combinations of SNRs and reverberation times. Because of this, the relatively low probability recall for both languages found in this experiment is expected. The important point to be made here is that the effects of reverberation and SNR (or lack thereof) cannot be fully explained by floor effects, even though the recall scores were relatively low, because the conditions clearly differed statistically and longer reverberation time had a positive effect at low SNRs. This is not compatible with the influence of a floor effect.

The interaction between SNR and reverberation time is arguably the most intriguing finding of the experiment reported here. Klatte et al. (2010) did not find an interaction between reverberation time and background noise on speech perception, but reverberation time was not varied in their listening comprehension task. There was no effect of reverberation time for speech perception of words presented against a silent background, but reverberation time impaired speech perception when background sound was present. Also, for third grade children, listening comprehension was found to be impaired by background sounds, but reverberation was not varied for listening comprehension. The findings in Klatte et al. (2010) clearly diverge from the results reported here, however, in view of the beneficial effects of a longer reverberation time. In particular, the children in the current experiment performed better in the +3 dBA condition with long reverberation time compared to the short reverberation time.

One possible explanation for this finding is that a long reverberation time assists the speech perception process and recall when the SNR is low because one hears the words for a slightly longer time. Perhaps, with more time available, the speech perception process is less resource costly, whereby more resources are available for storage and recall. Conversely, when SNR is high, longer reverberation time has only a signal distorting, rather than assisting, effect. A longer reverberation time in a classroom might therefor assist word recall for children when SNR is low compared to a higher SNR. This explanation is consistent with the finding that pathological language difficulties have their basis in a temporal processing deficit, and slower presentation times of the to-be-understood material can be particularly assisting in these populations (De Martino et al., 2001). One possibility is that, when the language perception process is impaired, either by person-specific cognitive skills or by external environmental factors like low SNRs, a somewhat longer presentation time of the to-be-perceived material facilitates the temporal integration of the speech signal, which makes it easier to understand and subsequently remember. This hypothesis is speculative, but may pave the way for future investigations into the potential benefits of a longer reverberation time—at least within some populations like children and people with undeveloped language skills.

## Author Contributions

AH writing parts: Introduction and Discussion. MP and SH writing parts: Statistical Analysis. EP writing parts: Materials and Methods. SH writing parts: Statistical Analysis and parts of Introduction. RL writing parts: Commentary. PS writing parts: Design and Commentary.

## Conflict of Interest Statement

The authors declare that the research was conducted in the absence of any commercial or financial relationships that could be construed as a potential conflict of interest.
